# The Perfect Match! A Review and Tutorial on Issues Related to Matching Groups in Investigations of Children with Neurodevelopmental Conditions

**DOI:** 10.3390/brainsci13101377

**Published:** 2023-09-27

**Authors:** David Messer, Lucy A. Henry, Henrik Danielsson

**Affiliations:** 1 Childhood and Youth Research Cluster, Faculty of Wellbeing, Education & Language Studies, Open University, Milton Keynes MK7 6AA, UK; 2 Department of Language and Communication Science, City, University of London, London EC1V 0HB, UK; lucy.henry.1@city.ac.uk; 3 Department of Behavioural Sciences and Learning, Linköping University, 581 83 Linköping, Sweden; henrik.danielsson@liu.se

**Keywords:** group matching, language acquisition, group comparisons, neurodevelopmental conditions

## Abstract

Research concerned with children and young people who have neurodevelopmental disabilities (ND) in relation to early language acquisition usually involves comparisons with matched group(s) of typically developing individuals. In these studies, several important and complex issues need to be addressed. Three major issues are related to: (1) the choice of a variables on which to carry out group matching; (2) recruiting children into the study; and (3) the statistical analysis of the data. To assist future research on this topic, we discuss each of these three issues and provide recommendations about what we believe to be the best course of action. To provide a comprehensive review of the methodological issues, we draw on research beyond the topic of early language acquisition. Our overall aim is to contribute to research that considers questions about delay or differences in development patterns of development and about identifying potentially causal variables.

## 1. Introduction

Considerable progress has been made in understanding the language development of children and young people with neurodevelopmental conditions and disabilities over the last 50 years. Many of the research studies that have produced this progress have involved investigations comparing neurodevelopmental groups with typically developing children and young people. Consequently, matched group comparisons are frequently used to address two key research questions.

The first of these question concerns whether children with neurodevelopmental conditions are performing as expected according to some index level such as chronological or mental age. Sometimes this question is extended to ask whether the pattern of development is *delayed* or *different*, and the answer to this question has implications for understanding and intervention [1,2], as well as for the provision of appropriate support and accommodations to maximise the young person’s ability to thrive. Children with neurodevelopmental conditions (NC) can be said to be *delayed* in their development if they do not match their peers of the same chronological age on a particular language skill or task, but keep pace with peers of an equivalent mental age on that skill or task (see the developmental model in [2,3]). In other words, language or other forms of development are proceeding along expected lines, and perhaps through similar stages, only more slowly, and, further, development may not reach the highest possible end performance levels. Alternatively, children with NC may have *different* performance to that of peers of a similar mental age on a particular skill or task, they may, in fact, exceed or be below what is expected for their mental age, and/or they may carry out the task or skill differently. In all of these cases, the researcher usually concludes that the development of a group with NC is different from the comparison group (see the difference model [4], which can be ‘positive’, i.e., better than expected performance, or ‘negative’, i.e., weaker than expected performance).

In practice, it can be difficult to establish that performance across all language abilities is delayed, and sometimes this question is restricted to a subset of abilities. Additionally, findings can sometimes support a combination of delayed and different performance across a range of abilities (e.g., [5]) or variations in the magnitude of group differences depending on the type of ability assessed [6]. It is worth noting that a non-significant difference between the TD and NC groups, which supports a delayed pattern of development, involves the usual difficulties associated with the interpretation of non-significant findings.

The second key question concerns whether we can identify processes, attributes or circumstances that are associated with the presence of NC or the language abilities of those with NC. This allows researchers to begin to identify variables that might make a causal contribution to any difficulties with language acquisition. These investigations are usually regarded as the first step towards a fuller investigation of causality.

Therefore, group comparisons are at the heart of research into NC, with conclusions qualified by the nature of the comparison group or groups chosen. However, in many ways, this research is attempting the impossible, trying to make two different groups with different abilities and life experiences as similar as possible. There have been several previous reviews considering how best to select and carry out research with comparison groups from a range of perspectives [7,8,9,10], and referring to these publications is recommended. However, these previous reviews have not covered all three of the topics we consider here.

In this review, we will use the term ‘children’ to refer to both children (in the primary school age range) and young people (in the secondary school age range) for simplicity and brevity. We also use the term neurodevelopmental conditions to refer to any group of children who have been identified as having learning disabilities, a difficulty or challenge with thinking and reasoning and/or adaptive behaviour (e.g., Down syndrome, specific learning disabilities such as dyslexia, and intellectual disabilities where IQ and adaptive functioning are below 70), and ‘TD’ to refer to groups of children who are showing typical development. That said, many of the recommendations are also valid when some form of matching and comparison are used with other groups. Additionally, we use the term ‘individual ability’ to refer to the variable used to match groups, and ‘specific skill’ to the language abilities that are compared across groups and are usually the dependent variables. In parts of this review, we refer to investigations that do not directly concern language acquisition, but hope the example serves to illustrate the point that we wish to make.

The three major sections of the review correspond to three major issues that need to be addressed by researchers. The first section considers the choice of variables on which to match and presents an evaluation of the most common forms of matching NC and TD groups. The second section is related to recruitment issues, including statistical checks on the equivalence of the groups. In the third section, we review statistical methods that are used in group comparisons. Following these sections is an overview, together with a summary of our recommendations. Our focus does not concern the issues associated with the assessment of language acquisition and development; however, our discussion of standardised tests and specific abilities are relevant to this topic.

In considering these topics we are not providing a recipe book that will result in the perfect investigation, but rather discussing a range of issues that help inform decision making. The challenge for investigators is to think through what research questions they wish to address, and how to best allocate their resources to minimise effects that could invalidate their findings. All this is a matter of judgement, such that greater awareness of different possibilities and potential pitfalls related to these three topics will increase the quality of research into NC.

## 2. Group Matching Using Chronological Age, Developmental Level or Specific Abilities

An appropriate design is of fundamental importance to address research questions and ensure confidence in the findings or, as Shadish et al. [11] emphasise, “the primacy of control [is] by design” (p. 105). Thus, it is important to understand the advantages and problems of variables that often are used for group matching. We consider these issues in three sub-sections: What Variables Are Used for Matching and What Can Different Forms of Matching Tell Us?; The Measurement of CA, General Developmental Level and Individual Abilities; and Concerns about Matching Groups Using Standardised Test Scores.

### 2.1. What Variables Are Used for Matching and What Can Different Forms of Matching Tell Us?

The following variables are often used for group matching: *chronological age* (CA); *general developmental level*, which is often assessed by standardised tests to give a mental age (MA) estimation; and *individual abilities*, such as a memory. The choice of variable depends on the research questions and characteristics of the NC group. All these forms of matching can be used to investigate target language skills in NC groups (i.e., the dependent variable in many studies). Matching on the basis of CA can answer questions about whether a specific skill in the NC group is age-appropriate, that is, whether it is at a level above, similar to or below that of children who have a similar chronological age. This form of matching has declined in use, partly because of the limited information it can provide about NC. Matching on the basis of general developmental level (e.g., intelligence test scores) provides the same information, but in relation to general cognitive level. For example, a researcher might investigate whether vocabulary in an NC group is on a par with, better than, or below a measure of their general intelligence. Matching on the basis of an individual ability such as memory addresses the same question about whether a language ability is higher than, similar to or below what would be expected in TD groups who have the same individual ability (i.e., memory). This latter type of matching is often appropriate when the NC group has a ‘spikey’ (i.e., very uneven) profile of abilities, so that matching on the basis of general developmental level is likely to be misleading.

Matching on the basis of general developmental level and on individual abilities can also be used to address questions about language delay or difference [1,2,12]. If there is no difference between the NC and TD groups matched in these ways, this suggests delayed and similar language development in the NC group. If language performance is not the same, this suggests different development in the NC group. CA matching usually does not allow direct investigation of delayed/different development as matching in this way will simply tell us the groups are not the same (though in some circumstances the developmental trajectories approach (see the section on this topic) can be used to answer question of delay/difference with CA-matched groups based on the shape of the growth curves).

It should be noted that all three forms of matching can help to identify potential causal variables in relation to language delays. If the specific target skill is significantly weaker in the NC than in the TD comparison group, this suggests that the skill could play a causal role in language acquisition in the NC group. However, it is important to note that some form of correlation or regression analysis to investigate whether performance on the target skill is related to language acquisition of the NC group is especially useful; for example, investigations have shown that phonological awareness is related to reading ability in dyslexia. Often, there is a reluctance on the part of researchers to carry out these analyses when the individual ability is part of the criteria for inclusion. However, we have not found convincing arguments to support this position.

Another useful, but rarely used, analysis is to determine the proportion of the NC group who have low abilities involving the target skill (e.g., the proportion who have memory abilities that are more than one standard deviation from the mean). If nearly all members of the group have a low score on the skill or ability, then this suggests a close and potentially causal relation to the language or other features of the NC. However, if an appreciable proportion of the NC group does not have a low score, then this suggests that the variable does not play a causal role, or that it plays a causal role only for some of the NC group. Therefore, additional analyses of relationships and proportions are likely to further our understanding, and we recommend researchers consider reporting these figures when any form of matching is used.

*Summary.* CA, MA and Individual Abilities are the variables usually used for group matching. CA matching can tell us about the abilities of a NC group in relation to what might be expected of children who have a similar age. The other forms of matching can address questions about whether the NC group has abilities different from what might be expected according to a general level of development (MA) or an individual aspect of development. All forms of matching have the potential to help identify causal variables.

### 2.2. The Measurement of CA, General Developmental Level and Individual Abilities

Chronological age can usually be measured easily and accurately, making this one of the most straightforward ways of matching between NC and TD groups. General developmental level in an NC is often assessed using a standardised test (e.g., Stanford-Binet-5; [13]), which can provide a summary score involving the estimation of mental age (MA) or an ‘age-equivalent’ score. This score gives the age at which the abilities of a child with NC are judged to correspond to the average abilities of children with TD. Ideally, the TD group should also be given the same standardised test, but sometimes their chronological age is used as a proxy for general ability. This will be a less accurate form of matching, because individual children’s chronological ages will rarely correspond exactly with their mental ages.

Although matching on the basis of general ability level often involves intelligence tests, other tests that provide summary scores can be used (e.g., motor abilities). We believe standardised tests of adaptive abilities that involve assessment of the extent to which children can carry out practical activities such as daily living skills, socialisation skills, communication and functional language (e.g., Vineland Adaptive Behaviour Scales 3, [14]) should be used more often for matching. These tests usually provide a similar set of scores to standardised assessments of cognitive ability (raw scores, standardised scores). There is likely to be increasing attention paid to measures of adaptive behaviour as a way of matching groups given that adaptive behaviour has been given more emphasis in the criteria for the identification of intellectual developmental disorder (intellectual disabilities) in DSM-5 [15,16], and because of the relevance of adaptive behaviour to everyday living. However, there is the potential for children to have different scores on mental age and adaptive behaviour, which could render matching very complicated, and there is the complication that communication skills are assessed as an adaptive behaviour.

In some research, rather than collect data from a matched TD group, data from the norms of the test are used. In this way, it is possible to investigate the profile of abilities in the NC group, compared to what has been measured in a typical sample using a simple test of difference. A considerable advantage of this approach is that the sample for standardisation is likely to be larger than that used in many research studies. However, it needs to be kept in mind that population-level standardised scores can change over time, e.g., [17], meaning that test scores based on older standardised samples may underestimate or overestimate differences between NC and TD groups. Furthermore, the samples used for the norms might not be comparable to the group with NC in terms of socio-economic status, mental age, or other important variables.

Sometimes, matching is based on a standardised test of a *single* individual ability (e.g., general non-verbal reasoning such as Raven’s Progressive Matrices; [18]), which is thought to provide an indication of the children’s *general* level of development. Often, these tests are quicker to administer than a general intelligence test. There can be justification for using a more specific test to assess general developmental level if the NC group has an uneven or ‘spikey’ profile of abilities, which makes a summary score from an intelligence test problematic (e.g., William’s syndrome).

Matching on the basis of individual abilities has become more popular, partly because of concerns about matching on the basis of standardised tests of general developmental level (see next section). Confusingly, non-verbal reasoning (and occasionally other) measures have been used for individual ability matching [19]. As just noted, these same abilities have also been used to match according to general ability level. Consequently, researchers need to be clear about whether they are using a more specific test, as a proxy for general developmental level, or whether the test is being used because there is a wish to match on an individual ability.

Another rationale for matching according to an individual ability is that it can be used to control for the influence of this ability on a specific skill. An example of this is where a group with intellectual disability and a TD group were matched on the basis of verbal fluency (the number of words produced about a category). This meant that the differences in semantic networks between groups were unlikely to be due to simple differences in fluency, and more likely to be due to other causes such as language environment or cognitive skills [20].

Often, matching on the basis of an individual ability is chosen because the NC group have a spikey profile of abilities. In such cases, the measure of individual ability could be a strength or a challenge in the NC group. As researchers are usually interested in whether a dependent variable is lower or higher than the usual abilities of a group of children with NC, the most appropriate design often involves matching on the basis of the abilities which are a strength. Consequently, it is important to make sure the chosen individual ability is relevant to the research question being addressed.

An unusual and innovative measure for individual ability matching is to use a TD sample to develop two versions of a task, so that the two versions produce equivalent performance [7]. Importantly, these two versions of the task are carefully designed to differ on a dimension thought to be important in the NC group. The researcher can then examine whether an NC group shows different performance levels on the two tasks. For example, autistic individuals carried out a social and a non-social picture-matching task that the researchers had established to be equally challenging for TD groups. This enabled the researchers to see whether an autistic group performed more strongly on the non-social picture-matching task [21]. Although most individual ability matching involves the use of standardised tests, the example here shows this does not always have to be the case.

*Summary.* The measurement of CA is usually straightforward and easy. Similarly, using standardised tests of MA or tests of individual abilities to assess developmental level does not usually present any technical difficulties. Rather, the difficulty is making sure that the form of matching relates to the research question being asked and takes into consideration the profile of abilities in the NC group, something we consider in more detail in the next section.

### 2.3. Concerns about Matching Groups Using Standardised Test Scores

Some disadvantages of obtaining an assessment of developmental level from a standardised intelligence test were identified nearly a century ago [22]. These problems include the fact that these tests are based on several abilities (e.g., expressive vocabulary, visuospatial reasoning, memory), so different children can have different strengths and challenges related to these abilities. As already noted [7,23,24], there is the possibility that children matched on the basis of a summary score such as MA will not, in fact, be matched on any of the component abilities that contribute to the summary score [25].

A related concern about general developmental level comparison can be illustrated by the following example that involves an investigation of whether there are greater challenges with executive functioning in children with developmental language disorder than in a TD group of a similar level of *general* ability. If the two groups are matched by MA from an intelligence test, this could be problematic. This is because some groups with NC have a spikey profile, as in the case with developmental language delay. Consequently, it is likely that the language disorder will result in lower levels of language-related abilities, but other abilities will be largely unaffected; this raises questions about what could be considered a general level of development in such children, as language-related abilities are usually an important contributor to the calculation of MA. Consequently, a measure of general developmental level can give an unrepresentative picture of the children’s abilities; a MA score of children with developmental language disorder is likely to be somewhere between the scores in areas of strength (e.g., non-verbal reasoning) and their areas of challenge (i.e., language). Thus, researchers need to make sure their research question can be addressed by the ability on the basis of which they choose to match groups. One solution to this problem has been to use non-verbal assessments of cognitive functioning and/or intelligence such as matrix tasks, and to additionally report data controlling for both verbal and non-verbal abilities for children with language disabilities or other developmental conditions where language is a potential confounding factor [26].

There also are concerns that when individuals with severe NCs are matched to a TD group according to MA or an individual ability, the TD group will be very much younger: sometimes many years younger. As a result, the two groups are likely to have very different social and educational experiences, which could affect the findings. For example, the greater life experience of the NC group can result in the further development and maturation of some abilities; receptive vocabulary is an ability that often shows such effects in NC [27]. The recruitment of another group with a different NC could help address this issue [28], but often this option requires considerable extra resources.

Another issue is the range of standardised assessments that are used in research. For example, in a meta-analysis of executive functions in individuals with intellectual disabilities [29], it was found that, of the 15 studies selected, 10 different standardised intelligence tests were used for MA matching. Even if these tests are relatively comparable, they are not identical, so it is difficult to draw firm conclusions when almost no investigation has employed a similar assessment. Consequently, if findings are inconsistent, it is difficult to identify the reason for this.

When using standardised tests, it is wise to check the growth curves of the ability being measured. A study of sub-test scores from commonly used intelligence tests checked how consistently these sub-test scores improved in relation to age, using normative data tables from the relevant test batteries [24]. It was noted that linear improvements in test scores with age were not always present; sometimes, there were plateaus or periods of sharper improvement. The authors recommended matching on the basis of sub-test scores that are linear, within the range of MA relevant to the study.

*Summary*. Although calculations of MA from standardised tests often appear to be an appropriate way to match on the basis of general developmental level, considerable care needs to be taken to ensure that this form of matching is appropriate for the profile of abilities in the NC group and the research question being addressed.

*Overview.* CA is an easy-to-obtain and valid measure. However, researchers need to consider whether matching according to CA will add to our understanding of groups with NC, as such comparisons can result in somewhat predictable group differences. Thus, in many circumstances, this form of group comparison is of limited value. Using an assessment of developmental level is a way of matching TD and NC groups, and this can provide useful information about whether language abilities are lower or higher than would be expected based on the child’s general level of functioning. Matching on the basis of developmental level has many issues that need to be thought through, especially in relation to the research question that is being addressed. There are concerns about general assessments of ability being based on several different abilities, especially when there are spikey profiles of abilities in NCs. There also are concerns about the psychometric properties of standardised scores and about potentially large chronological age gaps between TD and NC groups when matching on the basis of general ability level or individual ability. Furthermore, although general ability level matching provides information about delayed or different patterns of development, interpreting findings about potentially causal variables can be problematic. Matching TD and NC groups on specific abilities can go some way towards dealing with some of these concerns, and is desirable in groups with spikey profiles. It enables researchers to investigate whether children with NC are showing relative strengths, relative challenges, or performance at the expected level according to this more specific ability. Usually, it makes sense to match on an ability in the NC group which is a strength.

## 3. Recruitment: Inclusion Criteria, Matching Procedures, and Testing Group Equivalence

In this section, we discuss: (i) inclusion criteria in relation to the recruitment of children with co-occurring conditions and issues about subgroups; (ii) procedures for matching the groups; and (iii) checks on group equivalence.

### 3.1. Recruitment: Inclusion Criteria

Before recruiting a group of children with NCs, a decision needs to be made about inclusion criteria, especially in relation to those with co-occurring conditions. There can be a proportion of children with co-occurring conditions associated with NCs. For example, 18–50% of autistic children have an additional intellectual disability [30,31,32], and other co-occurring conditions such as ADHD are very common [33]. The absence or presence of co-occurring conditions is known to significantly affect the strengths and challenges of those included in NC groups [34,35]. In addition, care should be taken with the exclusion of children when matching, as there is the possibility that excluding children with NC, if no TD match is found (e.g., those with low standardised scores), could result in an unrepresentative sample [36].

We believe the inclusion or exclusion of co-occurring conditions should be decided in relation to the type of question being asked. If the question is more theoretical, about the characteristics of a particular form of NC, then it is appropriate to have a sample without co-occurring conditions. However, there is then a danger that findings from such a sample will not be generalisable to the whole population with that particular NC if the ‘pure’ condition is relatively rare. If one is more concerned with practical issues, such as support, accommodations, or interventions, then it is appropriate to include all individuals with co-occurring conditions. Attention should, however, be paid to the possibility that the co-occurring conditions could be confounding factors, or be partly responsible for the outcomes identified in the more general sample. Similar arguments can be made about matching the sex of NC and TD groups, as males are often more prevalent in NC groups. This can be resolved by deciding whether the TD comparison group should aim to represent the general population or be matched on relevant characteristics related to the target skill of interest.

Lombardo et al. [34] discussed a related issue, which is the presence of subgroups within the same NC clinical classification. They argued, in relation to autism, that the presence of subgroups with different profiles of abilities could affect findings when comparisons are made with matched groups. For example, if one subgroup is more able than the TD group, and another subgroup is less able, this may produce no overall significant difference between the NC and matched TD groups, which is a potentially misleading result. Lombardo et al. also made the point that comparisons using small samples can result in different findings between research investigations, due to each study having a slightly different composition of subgroups. Consequently, it is desirable to look at the variation in the NC sample and look for the presence of different clusters or subgroups (see also [37]). Because of these issues, Lombardo et al. [34] strongly argue for the use of large-scale studies in terms of both numbers of participants (>1000) and the number of assessments. Clearly, this is desirable, especially when there are suggestions that subgroups are present within a group, but it is also the case that such research requires collaboration and large-scale funding. Related to this issue, it has recently been argued that researchers should provide more detail about the variability of samples, for example, by providing figures about individual scores in addition to measures such as confidence intervals ([38]; also, see Section 4. Statistical Analysis of Group Differences).

*Summary.* We suggest that decisions about including co-occurring conditions be made in relation to the research questions being addressed and whether the focus is theoretical or practical. In addition, researchers need to be alert to the presence of sub-groups within NC samples.

### 3.2. Recruitment: Procedures for Matching

Usually, it makes sense to recruit the NC group first, as they are likely to be more difficult to locate. If the NC group is recruited first, the researchers can individually match children with TD to each child in the NC group. Ideally, the scores of matched children should be exactly the same on the relevant matching variables (e.g., CA or MA); however, often, this is not feasible. Another possibility is to use a sequential design, which involves carrying out group comparisons as data are collected, while taking account of Type 1 errors (i.e., accepting a difference between groups when there is none), thereby making the comparison process more efficient ([39]).

When an exact match is not possible, it is difficult to estimate the acceptable degree of difference between the scores. Scores on standardised tests are usually calibrated to identify differences in typical development over 3–4 months or more. Thus, it is sometimes argued that any difference between the matches below this length of time is acceptable, as the test does not make finer discriminations. However, smaller differences between matches could be problematic if the chosen matching variable (e.g., MA raw scores) is strongly related to the target skill, i.e., the outcome variable. When an exact match is not possible, tests of group equivalence should always be carried out (see below), and, if appropriate, the matching variable included as a control variable in a multiple regression (see Section 4).

Matching is sometimes carried out by using propensity scores, a technique devised by Rosenbaum and Rubin [40]. A propensity score is calculated for each participant, often by multivariate logistic regression, from background variables that are thought to predict the probability of a participant being in the NC group. Usually, there is a need to recruit more TD individuals than NC individuals to facilitate this form of matching, as the system chooses pairs of cases that have similar scores. The aim of matching in this way is to minimise differences between the groups in background characteristics, so that group comparisons will only be affected by the independent variable. However, there are criticisms of the use of propensity scores, particularly regarding the effects of pruning a sample to obtain a good match, as this can create unrepresentative groups [41]. It has also been noted that this technique requires large samples [10], and that problems remain in terms of knowing which variables to use when calculating propensity scores, and when deciding the criteria used to identify matches. The use of propensity scores in relation to NC and language acquisition is rare, and this probably reflects the difficulties of the procedure and the uncertainties about its effectiveness.

For some variables, it is difficult to know whether to try to match groups using specific variables or to try to ensure that both groups are matched because they are recruited from similar geographic areas. A good example of this is socioeconomic status (SES; others include physical and mental health). SES is thought to influence development in two ways [42]. Higher-SES families have more resources, which may increase the likelihood that they have their child identified with a disability compared to lower-SES families, particularly if the NC is mild, rather than severe. Conversely, in lower-SES families, there is a higher risk of a range of genetic/biological, environmental, psychological, social and health factors. Matching groups on a specific variable such as SES is desirable, but research studies often do not have the resources to carefully match children on the basis of SES, and questions about SES can be perceived as intrusive by many parents. As a result, many TD groups are recruited from areas containing middle SES families. Where possible, good practice is to recruit the TD children from the same areas or pre-schools as the children with NC. Where this is not possible, recruiting samples of TD and NC children from similar geographical areas (or from pre-schools that are similar on other indices related to SES) is a good option.

When using standardised tests to match groups, decisions need to be made about whether to match on the basis of MA scores (mental age-equivalence scores in years and months), the summary raw scores from the test, or standardised scores. MA scores are designed to correspond to the age equivalent functioning level of a child, and can be lower or higher than chronological age. The measurement error associated with MA can vary between tests, so it is worth checking the sensitivity of the age equivalent values. Some authors, including Mervis and Klein-Tasman [8], argue against using mental age scores because they have poor psychometric properties when it comes to assessing the extent of any disability (e.g., when MA is very low, the sample for standardisation usually contains few relevant participants), and they are not on an interval scale (limiting statistical analysis). Furthermore, it is usually the case that the *same* age-equivalence score can be obtained from a limited range of different raw scores, making raw scores a more sensitive measure of ability. Thus, in most circumstances, we recommend using raw scores from standardised tests rather than age-equivalence scores, although it should be acknowledged that raw scores are also not on an interval scale.

There is also the possibility of matching on the basis of standardised scores or T-scores [8]. We strongly advise against this in most circumstances. The standardised score for an ‘average’ child of any age is 100. Consequently, two average-level children at two different ages will differ in the absolute level of their ability (the older child will be more developmentally advanced and have a higher raw score), but they will not differ relative to other children of their own age. As a result, both children will have a standardised score of 100. Thus, standardised scores need to be used carefully, as unlike MA or raw scores, they do not give an indication of the absolute level of functioning of a child. Rather, a standardised score gives an indication of the degree of any disability relative to chronological age. Researchers should also be very careful about using standardised, mental age and raw scores in the same analyses due to their different psychometric properties.

*Summary.* Exact matches on relevant scores should be attempted whenever possible. However, when this is not possible, testing of group equivalence should be conducted. Care should be taken that the different groups are recruited from areas that have similar socio-demographic profiles. In most circumstances, we recommend matching on the basis of raw scores from standardised tests.

### 3.3. Recruitment: Group Equivalence

Once the groups have been recruited, statistical analyses are often carried out to check that the NC and TD groups have equivalent scores on the matched variable(s), such as CA, MA, or another variable. There has been discussion about the best way to do this, and Mervis and Klein-Tasman proposed that the *p*-value from a statistical test comparing the groups should be 0.50 or greater, rather than simply being non-significant, as has sometimes been the practice [8]. However, Kover and Atwood [10] drew attention to the limitations of this approach. A *p*-value of 0.50 would mean that, on average, the groups are *not* matched 50% of the time, when the groups are in fact matched. The authors also pointed out that the significance level would be larger for small samples, with their associated low power. Kover and Atwood [10] recommended, rather than reporting *p*-values, reporting the effect size, in order to provide a standardised mean difference (i.e., Cohen’s *d*) and variance ratios (variance of NC group/variance of TD group). Although they admit that there is uncertainty about the thresholds indicating acceptable matching, they recommend that the closer *d* is to zero and the closer the variance ratio is to 1, the better.

It has also been argued that, as well as determining that groups are matched on the chosen variable, the variability in their scores and the shapes of the distributions of their scores on the matching variable should also be checked [9], i.e., groups should be matched on the basis of both the mean and variance. Note that this will be achieved automatically if participants have been individually matched on the chosen variable, but such concerns are relevant if individual matching has not been conducted.

**Figure 1:**
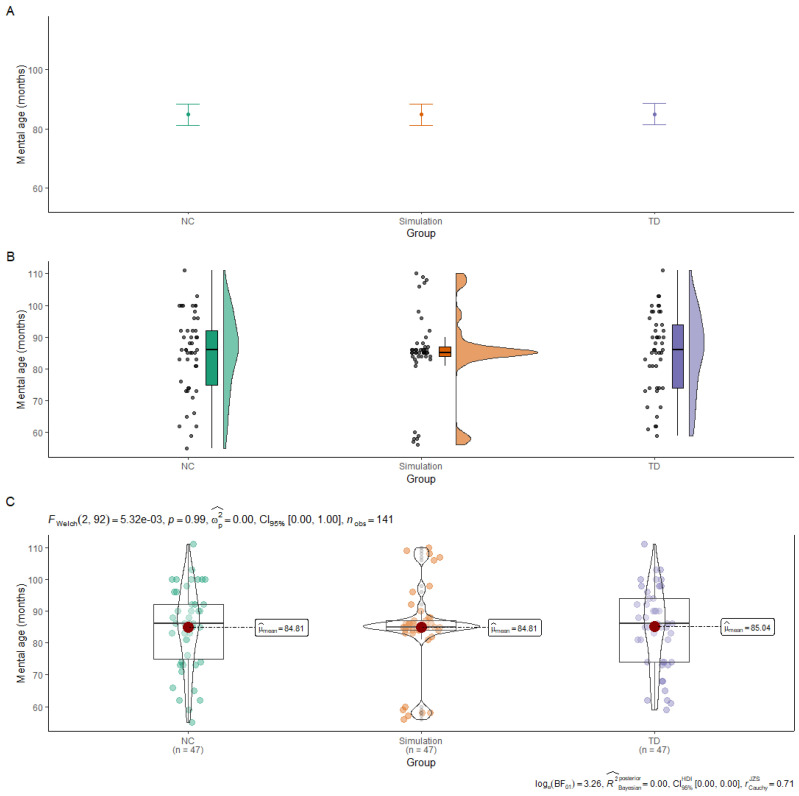
Three different ways, shown in panels (**A**–**C**), of plotting group characteristics in relation to whether the groups are equivalent (see text for a detailed explanation).

Figure 1 shows the limitations and advantages of different methods of reporting group equivalence and emphasise the benefits of displaying individual scores [38]. The NC and TD groups are from a sample who were carefully matched on the basis of both means and variance [43]. The *Simulation* group was created by us to provide an example of the limitations of using only means, standard deviations and significance values to test for group equivalence. Panel A, the top section of the figure, shows all three groups to have similar means and 95% confidence intervals; they appear equivalent, and there are non-significant differences (*p* = 0.99) between the groups. Panel B uses a Raincloud plot [44], which shows the individual datapoints (spaced horizontally so that all points are visible), a boxplot, and a half violin plot to show the distributions. As can be seen, the NC and TD groups are very similar, but the Simulation group has a very different distribution. Panel C gives a ggstatsplot [45] with the same information (the individual datapoints, a boxplot, and a violin plot); in addition, it also shows the mean and results from statistical tests on group difference. The statistical test can be customised; in this figure, the top right shows a Welsh *F*-test (Frequentist statistics) and the bottom right a Bayesian statistical equivalent.

*Summary.* We recommend that the reporting of tests of group equivalence between NC and TD samples include their mean scores, standard deviations, variance ratios, *p*-value, and effect size. Visual displays of the pattern of the scores of all participants is also desirable [38].

## 4. Statistical Analysis of Group Differences

In this section, different forms of statistical analysis related to group differences are reviewed: (i) analysis of group differences, covariation and regression; (ii) developmental trajectories; and (iii) fitting data to causal models.

### 4.1. Statistical Analysis: Group Differences, Covariation and Regression

*Simple Analysis of Group Differences.* Probably the most common method for comparing NC and TD groups is a simple between-groups test of difference. This method is well known, but we make three recommendations to help better understand the characteristics of the NC group. Firstly, if a significant difference between the TD and NC groups is interpreted as suggesting the target skill could be a causal factor in a disability (e.g., an NC group has lower verbal short-term memory than a TD group; [46]), then it is worth reporting whether this variable has a significant association with key indicators of the condition. For example, if executive functioning is found to be significantly lower in an NC group than in a TD group, it would be of interest to separately correlate executive functioning with language abilities in the two groups.

A second recommendation is that the percentage of individuals who are performing at a level that is appreciably below that of the comparison group should be reported. This can be done using z-scores or a cut-off score of one and/or two standard deviations below the TD mean. In this way, information is provided about the identified degree of difficulty in the NC group. Considerable variation has been reported in the extent to which different forms of executive functioning in an NC group (children with language difficulties) were below a score one or two standard deviations below the TD mean [26]. This type of reporting can alert researchers to the possibility of sub-groups within a syndrome [34], and, if relatively few in the NC group show appreciably lower performance than the TD group, this raises questions about the extent to which the variable is a key characteristic of the NC group. A related suggestion is to differentiate groups on a measure by using signal detection theory to identify the cut-point separating groups (sensitivity *Se* and specificity *Sp*), although, unfortunately, this does not appear to have been widely taken up [8].

A third recommendation concerns the inclusion of information about the group characteristics. Zhang et al. convincingly argued that the usual current practice of providing information about group differences using means and standard error bars (inferential uncertainty) can often provide a misleading impression of the variability in scores for a particular variable [38]. Therefore, they suggested the inclusion of figures displaying all of the data points for the groups included in the analysis (outcome variability; see Figure 1 for examples). They also made the important point that increasing the sample size reduces inferential uncertainty, but the variability will be likely to increase.

*Covariation and Regression.* It is possible when carrying out an analysis of group differences to statistically adjust for the effect of a variable using an Analysis of Covariance (ANCOVA) or by including these variables in the early steps of a multiple linear regression analysis. Usually, the same sets of variables that we have already discussed, namely CA, MA and specific abilities (e.g., a measure of memory), are candidates for these statistical adjustments.

Although analysis of covariance would appear to be a useful way to make statistical adjustments so that NC and TD groups are better matched on a potential confounding factor, this technique has been criticised. A major concern is that the groups should not differ on the covariate [7], yet often this statistical adjustment is made for precisely the reason that the groups are different. A further concern is that the covariate should be independent of group membership, and this is rarely the case [10,47]. For example, IQ or other measures of ability are very unlikely to be independent of TD/NC group membership. It is worth noting that covariation analyses make more stringent assumptions about the data than regression analyses (see below), and so the latter are often preferred as a method of adjustment.

Multiple linear regression, as the name implies, involves the assumption that there is a linear relationship between the independent and dependent variables. This assumption is rarely tested, but it should be [48]; logistic regression can be used when this assumption is violated. Multiple linear regression allows testing of group differences using dummy variables, with other variables, such as MA, being used as covariates. The technique is often used in studies of NC groups. It is important to ensure that the sample size is adequate, and for this there are a range of recommendations, with many suggesting there should be around 10–15 participants per variable. However, other ‘rules of thumb’ advise researchers to consider effect sizes and the number of predictors in determining minimum adequate sample sizes [49]. Another consideration is related to the number of control variables that are included in a regression analysis, with concerns that the inclusion of a multitude of variables can create complex interactions [50,51].

*Multi-Level Modelling*. An increasing number of publications appear to be using multi-level modelling to examine the variables related to the performance of groups with NC [43]. This technique enables a hierarchy of variables (e.g., individual ability, family characteristics, school membership) to be investigated in relation to an outcome measure, and has some advantages over regression analysis, such as estimating group effects simultaneously with the effects of group-level predictors. This is typically a good thing to do, but it should be weighed against the need for a larger sample size (compared to a regression analysis).

*Summary.* We recommend providing supplementary information about group differences such as relevant correlations and the degree of any impairment in performance. A useful feature of hierarchical regression analysis is that not only can group differences be examined, but covariates (control variables) can be included in the analysis.

### 4.2. Statistical Analysis: Developmental Trajectories

Another way to investigate differences between NC groups and TD groups is the analysis of developmental trajectories [52]. Although this technique is not widely used, it has several strengths and continues to attract interest. The technique can be related to growth curve modelling [53,54], and focuses on group differences in developmental progression, rather than focusing on differences at a specific age or ability level. Thus, an advantage of the developmental trajectories approach is that recruitment often can include a wider range of abilities. These data can be collected either cross-sectionally or longitudinally, or via a mixture of both [52].

A relatively simple developmental trajectory could involve the collection of information about a particular target skill (e.g., picture naming (see [55]) or short-term memory (see [56])) from NC and TD groups of different ages. Thus, growth in language can be charted across chronological age, mental age, or an individual ability such as memory. The growth curves that are produced can be analysed to address several interesting questions, as follows. (1) Is there a difference in the starting abilities between groups (the intercept of the two curves)? (2) Do any differences between NC and TD groups increase or decline with changes in an independent variable such as CA or MA? Such analyses can help to answer questions about developmental delay or difference in the NC group. If the trajectory of the NC group in relation to general developmental level is like that of the TD group, then this suggests a developmental delay [57]. Thomas et al. [52] outlined the way in which these findings can be used to determine whether an NC group shows delayed development, with parallel curves, or whether the curve for the NC group is different from the typical pattern. (3) Is there a relationship between the independent variable and the target skill? If this relationship is absent, it suggests that the independent variable is not related to language acquisition. This has been used to assess, for example, whether phonological awareness is the main predictor of decoding, and is a better predictor than, say, processing speed [52]. It is worth noting that such analyses have similarities with the regression analyses described in the previous section.

*Issues with Developmental Trajectories.* One practical problem with developmental trajectories is that they can require a large sample size, which may not always be practical for smaller-scale research projects. A further issue is related to the interpretation of any effects. It should be kept in mind that if there are non-overlapping and large differences in means, the differences in slopes between the groups can be problematic to interpret (similar to a non-interpretable interaction [58].

The choice of the ‘independent’ variable, such as chronological age, mental age, or an individual ability, when using a developmental trajectories analysis raises similar issues to those discussed in the previous sections. In particular, the reasons for choosing the independent variable need to be thought through in relation to the research questions being addressed. However, carrying out the analyses with several different independent variables (e.g., CA, MA, etc.) can provide new insights into the developmental progression of those with NC.

*Summary.* The developmental trajectory approach is very helpful when recruitment over a limited ability range is difficult. Furthermore, the method can provide comprehensive information about whether development is delayed or different. However, the technique is not suitable for low numbers of participants.

### 4.3. Statistical Analysis: Fitting Data to Causal Models

Structural equation modelling (SEM) enables researchers to test whether several identified theories about the causal factors involved in the development of a target skill are supported by the observed statistical associations between variables in the research data (e.g., which of several variables could influence the development of vocabulary) [59]. SEM can also involve assessing the degree to which a model accounts for the data in NC and TD groups, and if this is the objective, then consideration needs to be given to which variables are used to match the groups; otherwise, a difference between groups in causal structure could be due to a confounding variable (such a chronological age or SES). In SEM, it is assumed that relevant variables will have a normal distribution pattern, although this is often not assessed or reported. A good strategy is to use Directed Acyclic Graphs (DAGs) to specify the models of causation and then testing these against data with SEM [60]; however, DAGS are rarely used in relation to groups with NC.

An advantage of conducting SEM with an NC group and no TD comparison group is that the findings from such analyses can support one or other theory about the variables that influence the development of skills and abilities in children with NC. In this way, a direct comparison is avoided. This addresses concerns, often by groups who themselves have a neurodevelopmental condition, that the use of comparison groups can devalue the standing of a group with NC, and that NC groups should be studied in their own right and from their own perspectives [61]. However, if a TD group is not included, the researcher cannot draw conclusions about whether any associations are specific to the NC group.

Another way to investigate causal structures is regression-based path analysis [62]. This can be useful for more developed areas of research, where associations between key variables have already been established (e.g., phonological awareness with reading). This technique involves modelling paths between variables, which allows predictor variables to have indirect effects on key skills via another variable, i.e., the causal pathway can be indirect, and allows predictor variables to have effects only on certain ‘levels’ of a key skill, i.e., perhaps only affecting lower- or higher-scoring individuals. Such approaches (mediation and moderation analysis) help to reveal how and when associations between variables operate, but have not been widely used in relation to groups with NC.

*Issues with the use of Causal Modelling.* One important issue to consider is the large sample sizes that are necessary for SEM. There is wide variation in sample sizes that have been reported, with some studies reporting SEM using sample sizes of 58 in each of two groups [63], and others reporting larger sample sizes. There are several heuristics for determining sample size in SEM studies, which are typically based on (a) a fixed number (e.g., 100–200), (b) the number of participants per estimated parameter (e.g., 5–10), or (c) the number of participants per variable (e.g., 10). However, simulation studies [64] have shown that the required sample size also depends on many other factors, including the distribution of the variables, the amount of missing data, the reliability of the variables, and the strength of the relations among the variables. Because many of these factors are hard to estimate before a study, it makes it challenging to estimate the required sample size when planning a study. It is, however, possible to have a discussion in relation to these factors and end up with a reasonable sample size estimate [65].

Another issue is that SEM enables identification of the best fitting causal model from a set of possibilities according to the associations in the data. However, this does not establish that the model is correct—there could be other models, yet untested, that would provide a better fit of the data. Causal inference is very difficult to establish, and has many pitfalls that have been covered in more detail by other literature [66]. It should be noted that these pitfalls are mostly discussed in relation to advanced statistical methods, but they also apply to simpler analysis methods even if these have not been discussed to the same extent. Consequently, although we would argue that causal modelling provides very useful evidence about the relationship between variables, care must be taken with respect to overinterpretation of causal relationships, and the conclusions should be tested by other means such as randomised controlled trials.

*Summary.* Modelling of causal influences on NC abilities is an important method of analysis as positive evidence helps to provide a justification for experimental investigations in the form of interventions that target key skills. However, modelling often requires a large sample, and there is a need to have a sound rationale for the construction of different models.

## 5. Overview and Further Thoughts

It is apparent that the use of comparison groups in relation to children with NC involves considering many demands about methods and design. Despite the compromises that are inevitably needed, considerable progress in understanding language acquisition and development in NC has been made. In this article, we have discussed different ways of matching NC and TD groups, the recruitment of these groups, and statistical analysis. Our hope is that this will be of help to investigators who are planning to carry out research involving NC.

Although our focus has been on methods and designs that involve group comparisons, we are not arguing that group comparisons are always needed or desirable. Indeed, we wish to stress that for reasons of economy, and to avoid what are often negative descriptions of NC, there are good reasons for not including a TD group. Questions about potential causal variables can often be addressed without a TD group by investigating relationships within the group and by techniques such as SEM. Questions about delay or difference can be addressed when there is existing information about the developmental trajectory of a TD group [67].

When comparison groups are used, as in all investigations, research questions should drive the choice of groups, recruitment, and analysis. Matching NC and TD groups on CA is valid, reliable, and relatively easy. However, such comparisons can usually only be used to answer a limited set of questions. Analyses can show significantly weaker performance in the NC group, but this is usually expected. Furthermore, analyses using this design cannot usually answer questions about delay or difference and are of very limited value in identifying potentially causal variables.

Matching groups on the basis of general developmental level is particularly useful if the research question concerns whether the pattern of development is delayed or different. Furthermore, this form of matching can help to establish whether the NC group has a spikey profile, as in the case of a specific learning disability, with some abilities being at or near the levels of TD children, and other abilities being significantly stronger or weaker. Matching on the basis of an individual ability also can provide an answer to questions about whether the pattern of development is delayed or different. Evidence of significantly greater challenges on a target skill in a NC not only suggests a different pattern of development but also that the target skill might have a causal relationship with the disability. When previous research provides information about the profile of abilities in a NC, then general developmental level matching will usually be an appropriate for groups with a flat profile, whereas matching on the basis of an individual area of ability (that represents a strength) will usually be appropriate for groups with a spikey profile.

Given that adaptive behaviour has recently been included in the definition of intellectual disability, we expect that future research will match on adaptive behaviour, probably together with MA, CA and/or specific abilities. Such research is likely to provide a useful focus on practical everyday activities that may be of help to parents, carers, and professionals, and in developing the language and life skills of the individuals with NCs. It is possible to argue that a new focus on more general life skills is likely to be of considerable benefit to the wellbeing of individuals with NC, especially if this can also involve a focus on ability rather than deficit. This approach can also bolster our understanding of how best to support those with NC to thrive using optimal environments and adjustments that accommodate and minimise areas of challenge. However, care needs to be taken in relation to the use of summary scores of adaptive behaviour in NC groups suspected of having language difficulties because of the likely relationship between measures of adaptive communication and the language difficulties.

There are several practical issues that should be addressed in the recruitment of matched samples. Exact matches to individual children in the NC group should be made when possible. If this is not achievable, then group equivalence of the variables used for matching should be tested, with the reporting of the mean scores, standard deviations, variance ratio, *p*-value, effect size and a visual profile of their scores. Another consideration is the high incidence of co-occurring conditions in NC groups. Thus, investigators are faced with a choice of whether to be selective and only include children with no co-occurring conditions, or whether to include these individuals. We believe that this decision should be guided by the aims of the research in terms of whether it is better to have a sample where confounding variables are absent, or whether the desire is to understand more about the strengths and challenges of all individuals with a specific condition. Related to this is the need to monitor for the presence of subgroups.

We have argued that the simple examination of differences between matched groups often is of limited value in deciding whether a target skill potentially has a causal role in the NC. This is because even though a significant difference between groups in the target skill suggests the possibility of a causal influence, a non-significant difference between groups does not rule out this possibility. We believe that more convincing evidence can be obtained if a significant correlation or predictive relationship is detected between a target skill and a core dimension of the disability. It is advantageous to do this in both NC and TD groups, as there could be different associations in the two groups, although in some circumstances a TD matched group is not essential. Correlations and predictive relationships do not establish causality, but they provide useful clues about where to investigate further. This type of analysis will usually be better served by multiple regression and related analyses than the simple analysis of group differences, especially as regression analyses also can be used to examine group differences by using dummy variables. We also suggest that when possible, researchers should report the proportion of the NC group whose target skill is below the typical range. This can provide useful information of the extent of a link between the target skill and the disability, and be useful in terms of detecting the presence of subgroups.

The developmental trajectory approach enables the growth of language abilities in NC and TD groups to be compared and, consequently, usually provides an answer to questions about developmental difference or delay in relation to a greater age or ability range. However, researchers still need to think through their choice of ‘independent’ and ‘dependent’ variables in relation to their research question and the characteristics of the NC group. The testing of causal models in path analysis and SEM enables researchers to evaluate theories about causal influences in a non-experimental investigation. The disadvantage is that these analyses are likely to require large groups of individuals, and it needs to be remembered that although they can identify which theories about relationships or causality best fit the data, further research is needed to confirm these links.

Research involving NC groups is a difficult process, and identifying differences between NC and TD groups is crucial to answer a range of questions about language acquisition and other processes, particularly those about the identification of variables that potentially could play a causal role or could be the focus of intervention, support, adjustments, and accommodations. Greater understanding of selecting comparison groups, recruiting matched samples, and statistical analyses should help to accelerate the progress that already has been made in the understanding of a range of neurodevelopmental conditions. Furthermore, the new processes found in Open Science should increase the validity, transparency, and reliability of the findings of these investigations.

## Data Availability

Data is available on request from the first author.
